# Maternal Periconceptional and Gestational Low Protein Diet Affects Mouse Offspring Growth, Cardiovascular and Adipose Phenotype at 1 Year of Age

**DOI:** 10.1371/journal.pone.0028745

**Published:** 2011-12-15

**Authors:** Adam J. Watkins, Emma S. Lucas, Adrian Wilkins, Felino R. A. Cagampang, Tom P. Fleming

**Affiliations:** 1 School of Biological Sciences, University of Southampton, Southampton General Hospital, Southampton, United Kingdom; 2 School of Biosciences, University of Nottingham, Loughborough, United Kingdom; 3 Human Development and Health Unit, Faculty of Medicine, Institute of Developmental Sciences, University of Southampton, Southampton General Hospital, Southampton, United Kingdom; Brigham and Women's Hospital, United States of America

## Abstract

Human and animal studies have revealed a strong association between periconceptional environmental factors, such as poor maternal diet, and an increased propensity for cardiovascular and metabolic disease in adult offspring. Previously, we reported cardiovascular and physiological effects of maternal low protein diet (LPD) fed during discrete periods of periconceptional development on 6-month-old mouse offspring. Here, we extend the analysis in 1 year aging offspring, evaluating mechanisms regulating growth and adiposity. Isocaloric LPD (9% casein) or normal protein diet (18% casein; NPD) was fed to female MF-1 mice either exclusively during oocyte maturation (for 3.5 days prior to mating; Egg-LPD, Egg-NPD, respectively), throughout gestation (LPD, NPD) or exclusively during preimplantation development (for 3.5 days post mating; Emb-LPD). LPD and Emb-LPD female offspring were significantly lighter and heavier than NPD females respectively for up to 52 weeks. Egg-LPD, LPD and Emb-LPD offspring displayed significantly elevated systolic blood pressure at 52 weeks compared to respective controls (Egg-NPD, NPD). LPD females had significantly reduced inguinal and retroperitoneal fat pad: body weight ratios compared to NPD females. Expression of the insulin receptor (*Insr*) and insulin-like growth factor I receptor (*Igf1r*) in retroperitoneal fat was significantly elevated in Emb-LPD females (P<0.05), whilst Emb-LPD males displayed significantly decreased expression of the mitochondrial uncoupling protein 1 (*Ucp1*) gene compared to NPD offspring. LPD females displayed significantly increased expression of *Ucp1* in interscapular brown adipose tissue when compared to NPD offspring. Our results demonstrate that aging offspring body weight, cardiovascular and adiposity homeostasis can be programmed by maternal periconceptional nutrition. These adverse outcomes further exemplify the criticality of dietary behaviour around the time of conception on long-term offspring health.

## Introduction

Human and animal model data support an association between sub-optimal intrauterine environments, altered fetal growth and the predisposition to adult-onset disease [1], [2]. Development of the metabolic syndrome, characterized by hypertension, central obesity and insulin resistance, subsequently increases an individual's risk for later type 2 diabetes and cardiovascular disease. These studies further demonstrate that maternal nutritional challenges during critical periods of gestation impose distinct effects on homeostatic mechanisms resulting in long-term disease risk.

The majority of studies manipulating the maternal environment have examined the interaction between macro- or micronutrient intake during gestation on offspring development and health. In the rat, maternal food restriction (30–50% of *ad libitum*) during gestation and lactation has been shown to reduce offspring weight and pancreatic *β*-cell mass at birth [3], [4], induce glucose intolerance [5], hyperphagia, hyperinsulinemia, hypertension, and obesity in adulthood [6]. Similarly in rodents, the feeding of a maternal low protein diet (LPD) throughout gestation and/or lactation affects long-term offspring health. Here, significant changes in weight at birth [7], altered adiposity [8], [9], preferences for high-fat foods [10], insulin resistance [11], reduced pancreatic *β*-cell mass [12], hypertension and vascular dysfunction [13], [14] have all been reported.

However, there is now increasing evidence that maternal nutrition during the periconceptional period, and even prior to conception, may contribute to offspring disease risk [15]. Human epidemiological observations from the ‘Dutch Hunger Winter’ studies have shown that adults born to mothers exposed to poor nutrition during early pregnancy displayed increased early-onset incidences of coronary artery disease [16] and in men, increased prevalence of intra-abdominal obesity [17], when compared to adults exposed during mid- or late gestation. In the sheep, reductions in maternal food intake, or feeding of diets deficient in B vitamins and methionine during the periconceptional period (between 60 days preconception to 31 days gestational age), elevates fetal blood pressure, induces cardiovascular and renal abnormalities, impairs glucose tolerance and insulin resistance and increases adiposity in adult offspring [18], [19], [20], [21]. Recently, it has been shown that feeding a methyl deficient diet to rat dams, from 3 weeks preconception to 5 days following conception, resulted in altered glucose tolerance in male offspring [22]. Moreover, in the mouse, feeding a maternal LPD for 8 weeks prior to conception has been shown to increase fat deposition and alter digestive physiology in offspring [23].

Using our established mouse model, we have narrowed the window of sensitivity further, identifying the terminal stages of oocyte maturation, as well as the preimplantation period, as being critically sensitive to maternal nutrition. Maternal LPD administered exclusively during these stages elevated fetal and postnatal growth, induced hypertension and vascular dysfunction and altered behavioural profiles in the adult offspring [24], [25], [26].

There are few animal or human studies investigating whether deficient maternal nutrition around the time of conception contributes to the development of metabolic syndrome in aging animals. Such studies would determine the transitory or permanent nature of periconceptional programming processes. We have therefore extended our previous mouse studies [25], [26] to investigate central components of the metabolic syndrome, notably growth, cardiovascular phenotype, glucose and insulin homeostasis, adiposity and fat gene expression patterns in mouse offspring at 1 year of age. We find a clear link between maternal periconceptional nutrition and the development of the metabolic syndrome in offspring during aging.

## Materials and Methods

### Animal treatments

**Figure 1:**
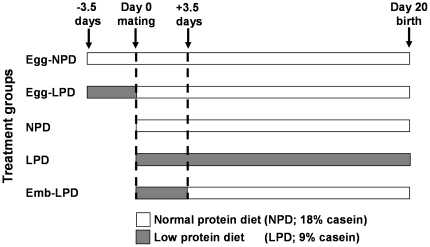
Diagram depicting the 5 dietary regimes used in this study. Females were fed either normal protein diet (NPD) throughout oocyte maturation (3.5 days prior to mating; Egg-NPD), low protein diet (LPD) exclusively during oocyte maturation (Egg-LPD), NPD though out gestation, LPD throughout gestation (LPD) or LPD exclusively during preimplantation development (3.5 days following mating; Emb-LPD).

All mice and experimental procedures were conducted using protocols approved by, and in accordance with, the UK Home Office Animal (Scientific Procedures) Act 1986 and local ethics committee at the University of Southampton under UK Home Office Project Licence PPL30/2467. Maternal dietary treatments and offspring postnatal analyses were conducted as previously described [25], [26]. Briefly, virgin female MF-1 mice (aged 7–8.5 weeks) previously maintained *ad libitum* on standard laboratory chow (Special Diet Services) were randomly allocated to one of five isocaloric dietary treatment groups, being fed *ad libitum* with free access to water, either low protein diet (LPD; 9%) or normal protein diet (NPD; 18%) (Table 1) exclusively during oocyte maturation (3.5 days prior to mating then chow for the duration of gestation; termed Egg-LPD and Egg-NPD respectively), throughout gestation only (termed LPD and NPD respectively) or exclusively during preimplantation development (from vaginal plug identification until 3.5 days; termed Emb-LPD) before being switched to NPD for the remainder of gestation (see Fig. 1). All females were individually coded and fed such that the experimenter was blinded to the dietary treatment each female received, and thereby the treatment group of the offspring. Nineteen litters of each dietary treatment group were generated with litter size adjusted at birth to a mean of 6 (3 males and 3 females, where possible) with all pups being returned to their own dam. From birth and weaning respectively, all dams and offspring were maintained *ad libitum* on standard laboratory chow (Special Diet Services) until time of cull. Offspring were randomly tail marked at weaning (1–3 bands with permanent marker) for subsequent weekly tracking of individuals. At 6 months of age, the majority of offspring were culled (selected by their tail marks) and their postnatal development and phenotype reported in detail previously [25], [26]. One remaining animal of each sex from 9–12 litters of each treatment was maintained further under the same environmental conditions until 52 weeks of age. To determine whether the males and females selected for analysis within the present study (1 year cohort) were representative of the larger cohort from which they were selected (6 month cohort), we compared offspring body weight (3 weeks and 6 months) and systolic blood pressure (SBP) at 21 weeks. Weight at 6 months of age was significantly elevated in Emb-LPD females within the 1 year cohort when compared to NPD females (Table 2; P = 0.023). However, no other significant differences were observed between treatment groups. Comparison of weight and SBP between the same treatment group within the 1 year cohort and 6 month cohorts revealed significant differences in SBP for Egg-NPD males and for NPD males and females (Table 3 and 4; P<0.05). Whilst parameters measured from the 1 year cohort were not identical to those of the larger cohort (Table 3 and 4), due to the persistence of changes in body weight (Table 2), a factor we previously identified as a major determinant and predictor of adult health, it was felt that the animals used within the present study sufficiently represented the larger cohort from which they came.

**Table 1:** Macro- and micronutrient composition of the diets used within the study.

	g/kg Inclusion	
	NPD	LPD
Starch Maize	425	485
Sucrose	213	243
Casein	180	90
Corn Oil	100	100
Cellulose	50	50
Mineral mix^1^	20	20
Vitamin mix^2^	5	5
DL-methionine	5	5
Choline Chloride	2	2
Gross Energy, MJ/kg	18.39	18.27

1 Mineral mix (AIN-76): (Special Diet Services).

2 Vitamin mix (AIN-76): (Special Diet Services).

**Table 2:** Comparison of body weight and systolic blood pressure (SBP) measurements from mice analysed within the present study.

		Egg-NPD	Egg-LPD	NPD	LPD	Emb-LPD
**Males**	**Weight at 3 weeks**	16.1±0.9	15.8±0.7	13.0±0.5	13.7±0.5	13.2±0.5
	**Weight at 6 months**	46.7±0.7	49.5±0.7	47.2±1.7	48.5±2	47.3±1.6
	**SBP at 21 weeks**	109.0±0.6	113.1±1.3	110.8±1.6	110.1±0.7	111.0±1.9
**Females**	**Weight at 3 weeks**	15.9±0.8	15.8±0.5	13.0±0.6	13.1±0.4	12.7±0.4
	**Weight at 6 months**	40.1±0.8	41.6±0.7	37.3±1	38.5±1.2	41.9±1.6*
	**SBP at 21 weeks**	105.8±1.5	110.3±2.0	110.7±1.7	113.6±1.4	111.9±2.0

Values are means ± SEM for 9–13, 11–12, 9–12, 8–10 and 9–11 Egg-NPD, Egg-LPD, NPD, LPD and Emb-LPD males and females respectively;

*P = 0.023 when compared to NPD females.

**Table 3:** Comparison of male offspring body weight and systolic blood pressure (SBP) measurements of mice analysed within the present study (1 Year Cohort) with the same treatment group from the larger cohort (6 Month Cohort) from which they were selected.

	Egg-NPD		Egg-LPD		NPD		LPD		Emb-LPD	
	6 Month Cohort	1 Year Cohort	6 Month Cohort	1 Year Cohort	6 Month Cohort	1 Year Cohort	6 Month Cohort	1 Year Cohort	6 Month Cohort	1 Year Cohort
**Weight at 3 weeks**	16.2±0.9	16.1±0.9	14.9±0.7	15.8±0.7	13.3±0.3	13.0±0.5	13.5±0.5	13.7±0.5	13.6±0.3	13.2±0.5
**Weight at 6 months**	47.1±0.7	46.7±0.7	46.3±0.7	49.5±0.7	48.1±1	47.2±1.7	47.1±0.8	48.5±2.0	48.9±0.6	47.3±1.6
**SBP at 21 weeks**	103.1±0.6	109±0.6*	111.1±1.3	113.1±1.3	105.6±1.1	110.8±1.6*	111.1±0.7	110.1±0.7	109.6±0.9	111.0±1.9

Values are means ± SEM for 55–56, 42–52, 38–53, 46–61 and 47–57 Egg-NPD, Egg-LPD, NPD, LPD and Emb-LPD males respectively for the 6 month cohort and 8–13, 11–12, 12, 7–8 and 11 Egg-NPD, Egg-LPD, NPD, LPD and Emb-LPD males respectively for the 1 year cohort;

*P<0.05 when compared to the same treatment group.

**Table 4:** Comparison of female offspring body weight and systolic blood pressure (SBP) measurements of mice analysed within the present study (1 Year Cohort) with the same treatment group from the larger cohort (6 Month Cohort) from which they were selected.

	Egg-NPD		Egg-LPD		NPD		LPD		Emb-LPD	
	6 Month Cohort	1 Year Cohort	6 Month Cohort	1 Year Cohort	6 Month Cohort	1 Year Cohort	6 Month Cohort	1 Year Cohort	6 Month Cohort	1 Year Cohort
**Weight at 3 weeks**	15.0±0.8	15.9±0.8	14.5±0.5	15.8±0.5	12.3±0.4	13.0±0.6	12.6±0.2	13.1±0.4	13.4±0.3	12.7±0.4
**Weight at 6 months**	40.2±0.8	40.3±0.8	40.3±0.7	41.6±0.7	39.5±1.1	37.3±1.0	39.9±0.6	38.5±1.2	42.8±0.7	41.9±1.6
**SBP at 21 weeks**	102.6±0.8	105.8±1.5	108.8±1.24	110.3±2.0	104.2±1	110.7±1.7*	110.4±1	113.6±1.4	112.1±0.9	111.9±2.0

Values are means ± SEM for 51–54, 50–57, 48–50, 48–50 and 48–49 Egg-NPD, Egg-LPD, NPD, LPD and Emb-LPD females respectively for the 6 month cohort and 10–12, 10–12, 12, 8–9 and 11 Egg-NPD, Egg-LPD, NPD, LPD and Emb-LPD females respectively for the 1 year cohort;

*P<0.05 when compared to the same treatment group.

### Systolic blood pressure

At 52 weeks of age, offspring SBP was measured by tail-cuff plethysmography as previously described [25], [26]. Briefly, mice were allowed to acclimatize to a room temperature of 27–30°C before four recordings were taken per mouse; heart rate was monitored as an indicator of stress [27].

### Organ allometry

At 52 weeks of age animals were deprived of food (*ad libitum* water access was preserved) for a period of 4 hours prior to being weighed and sacrificed via cervical dislocation. Body length (nose-anus and nose-tail tip) was recorded for the calculation of body mass index (BMI; g/cm^2^). Blood samples were removed via cardiac puncture and kept on ice to clot before centrifugation for 10 minutes at 10,000 g and 4°C. The serum was aliquoted and stored at −80°C. Liver, left and right kidneys, heart, lungs and brain were dissected, weighed, and snap frozen in liquid nitrogen prior to storage at −80°C. Mesenteric (surrounding the small intestine), retroperitoneal (from the perirenal capsules as well as that attached to the dorsal body wall near the kidneys), gonadal (surrounding epididymal and vesicular glands in males and the ovaries and uterus in females) and inguinal (subcutaneous depot anterior to the upper segment of the hind limbs) white adipose pads as well as interscapular (between shoulder blades) brown adipose tissue (IBAT) were removed, weighed and stored as above to assess offspring fat deposition.

### Serum glucose and insulin measurements

Serum glucose concentrations were measured using the Infinity™ Glucose Oxidase Liquid Stable Reagent kit (Thermo) according to manufacturer's instructions. All samples were analysed in duplicate against a standard curve (0–200 mg/dL) and a reference sample analysed in every assay to measure inter-assay variation (10.46%).

Serum insulin was measured using a mouse ultra-sensitive ELISA (DRG Instruments) according to manufacturer's instructions. All samples were analysed against a standard curve (ranging from 0–6.5 µg/L) and a reference sample analysed in every assay to measure inter-assay variation (1.77%).

### RNA extraction and RTqPCR for gene expression analysis

RNA was extracted from IBAT and retroperitoneal fat using the RNeasy® Lipid Tissue Mini Kit (QIAGEN, UK) according to manufacturer's instructions. On-column DNase I digestion was performed to remove contaminating genomic DNA. RNA was reverse transcribed to cDNA using the ImProm™II kit (Promega, UK) and a random priming strategy, according to the manufacturer's instructions. Intron-spanning primers used in this study are detailed in Table 5. For Real-Time PCR (RTqPCR), a mastermix was prepared containing 10 µl 2× Precision Mastermix containing SYBRgreen (PrimerDesign, UK), 1.2 µl primer mix (containing 5 µM each forward and reverse primers) and 7.8 µl water per reaction. Mastermix was aliquoted to 96 well PCR plates (Axygen, UK) in 19 µl volumes and 1 µl cDNA was added to sample wells (equivalent to 5 ng RNA) or water to control (no template) wells, giving a final reaction volume of 20 µl. All samples were run in triplicate. A calibrator sample was included on all runs to control for inter-assay variation. Amplification and detection was performed using a DNA Engine thermal cycler and Chromo4 Real-Time Detector and data was acquired using Opticon Monitor Version 3.1 software (BioRad, UK). A post-amplification melting curve confirmed the presence of specific products for each primer set. Male and female IBAT data were normalised to the expression of peptidylprolyl isomerase B (*Ppib*) and TATA box binding protein (*Tbp*) whilst the retroperitoneal fat data were normalised to the expression of phosphoglycerate kinase 1 (*Pgk1*) and succinate dehydrogenase complex, subunit alpha (*Sdha*). geNorm software [28] was used to determine these to be the most stable reference genes in the adult mouse fat tissues [29].

**Table 5:** Real-Time PCR primer details for gene expression studies.

Gene Name	Gene Symbol	Accession Number	Primer Sequences		Amplicon Length	Primer efficiency
			Forward Primer	Reverse Primer		
Uncoupling protein 1 (mitochondrial, proton carrier)	*Ucp1*	NM_009463.2	ggcctctacgactcagtcca	taagccggctgagatcttgt	84	1.99
Insulin receptor	*Insr*	NM_010568.1	ggattattgtctcaaagggctgaa	gagtcgtcatactcactctgattgtg	102	1.93
Adrenergic receptor, beta 3	*Adrb3*	NM_013462.3	cagccagccctgttgaag	ccttcatagccatcaaacctg	61	1.99
Insulin-like growth factor I receptor	*Igf1r*	NM_010513.2	ccagaagtggagcagaataatc	gccatgccatctgcaatct	89	1.98
Succinate dehydrogenase complex, subunit A, flavoprotein	*Sdha*	NM_023281	tgttcagttccaccccaca	tctccacgacacccttctgt	66	1.99
Phosphoglycerate kinase 1	*Pgk1*	NM_008828	tacctgctggctggatgg	cacagcctcggcatatttct	65	1.99
TATA box binding protein	*Tbp*	NM_013684.3	gggagaatcatggaccagaa	gatgggaattccaggagtca	90	1.94
Peptidylprolyl isomerase B	*Ppib*	NM_011149	ttcttcataaccacagtcaagacc	accttccgtaccacatccat	92	1.95

### Statistical analyses

All data (postnatal weights, SBP, organ allometry, serum insulin and glucose levels and fat pad gene expression) were analysed using a multilevel random effects regression model (SPSS version 18) as described previously [30], [31]. This allows the hierarchical nature of the data to be addressed, accounting for between-dam and within-dam variation when analysing different parameters from individual animals. Additionally, growth data were converted to Z-scores using SPSS prior to statistical analysis so that entire growth curves could be compared across treatment groups [26]. All data are adjusted for maternal origin of litter, gestational litter size and body weight effects where appropriate. Significant influences of gestational litter size and body weight on data are reported. Significance was assumed at P<0.05.

## Results

### Postnatal growth

**Figure 2:**
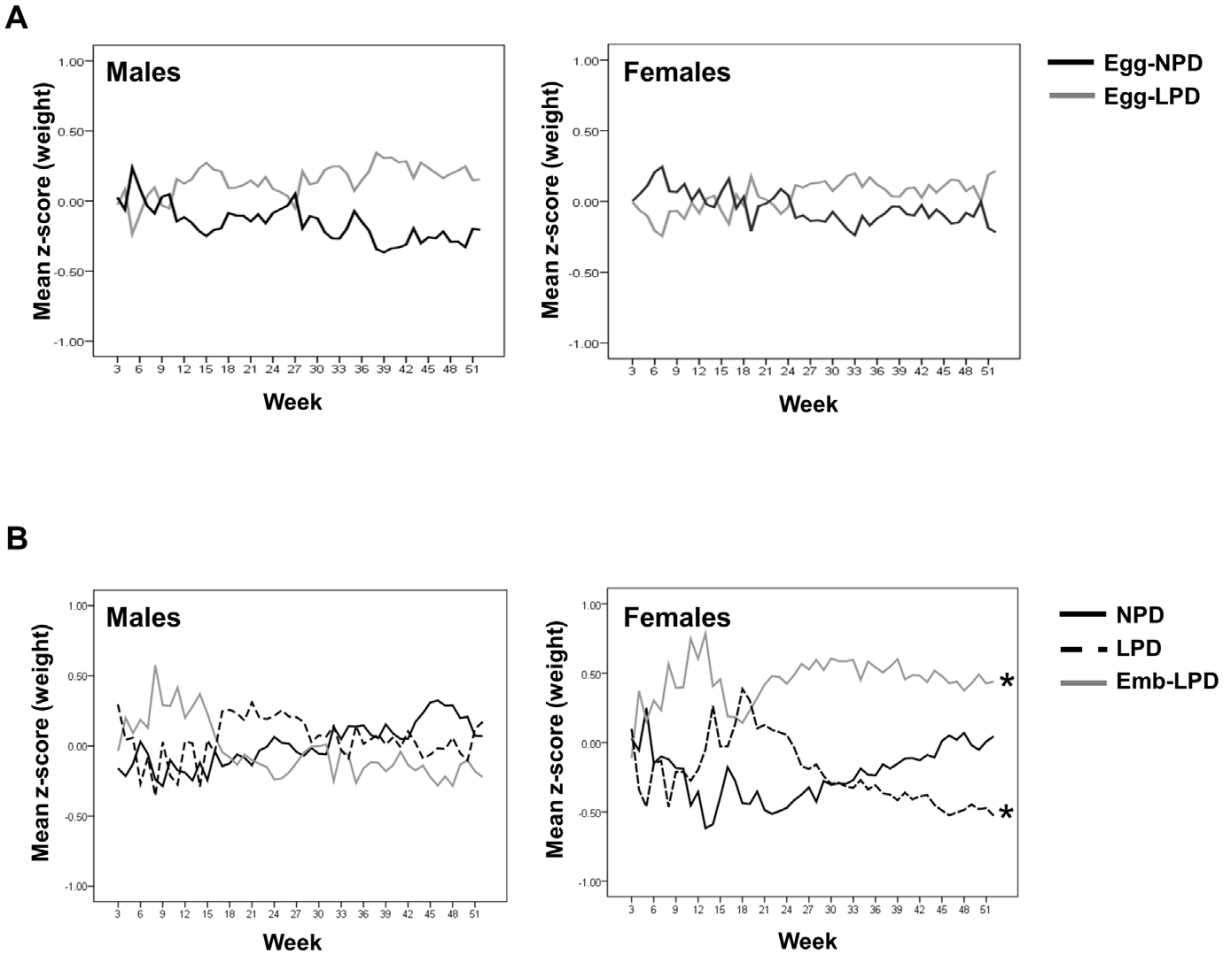
Offspring growth Z-score profiles in response to maternal diet. Effect of maternal LPD given either (A) exclusively during oocyte maturation (Egg-LPD), or (B) either throughout gestation (LPD) or exclusively during preimplantation development (Emb-LPD) on offspring growth Z-score profiles for up to 52 weeks when compared with offspring fed maternal normal protein diet (Egg-NPD and NPD respectively). n = 9–13, 11–12, 9–12, 8–10 and 9–11 Egg-NPD, Egg-LPD, NPD, LPD and Emb-LPD males and females respectively; *P<0.01.

No difference in postnatal body weight was observed between Egg-LPD and Egg-NPD males or females for up to 1 year of age (Fig. 2A), or between NPD, LPD and Emb-LPD males (Fig. 2B). LPD females were lighter than NPD females at 46 weeks, whilst Emb-LPD females were heavier than NPD females at 11, 12, 13, 14, 21, 22, 23, 26 and 28 weeks of age (P<0.05). Analysis of entire postnatal growth curves (Z-scores) showed that Emb-LPD females had an elevated growth profile, whilst LPD females had a reduced growth profile compared to NPD females (Fig. 2B; P<0.001 and  = 0.006 respectively). Analysis revealed gestational litter size to have a significant negative effect, such that for every increase in litter size by a single offspring, mean growth Z-score decreased by 0.069 (P<0.001); however, no differences in litter sizes were observed between treatment groups.

### Systolic blood pressure (SBP)

**Figure 3:**
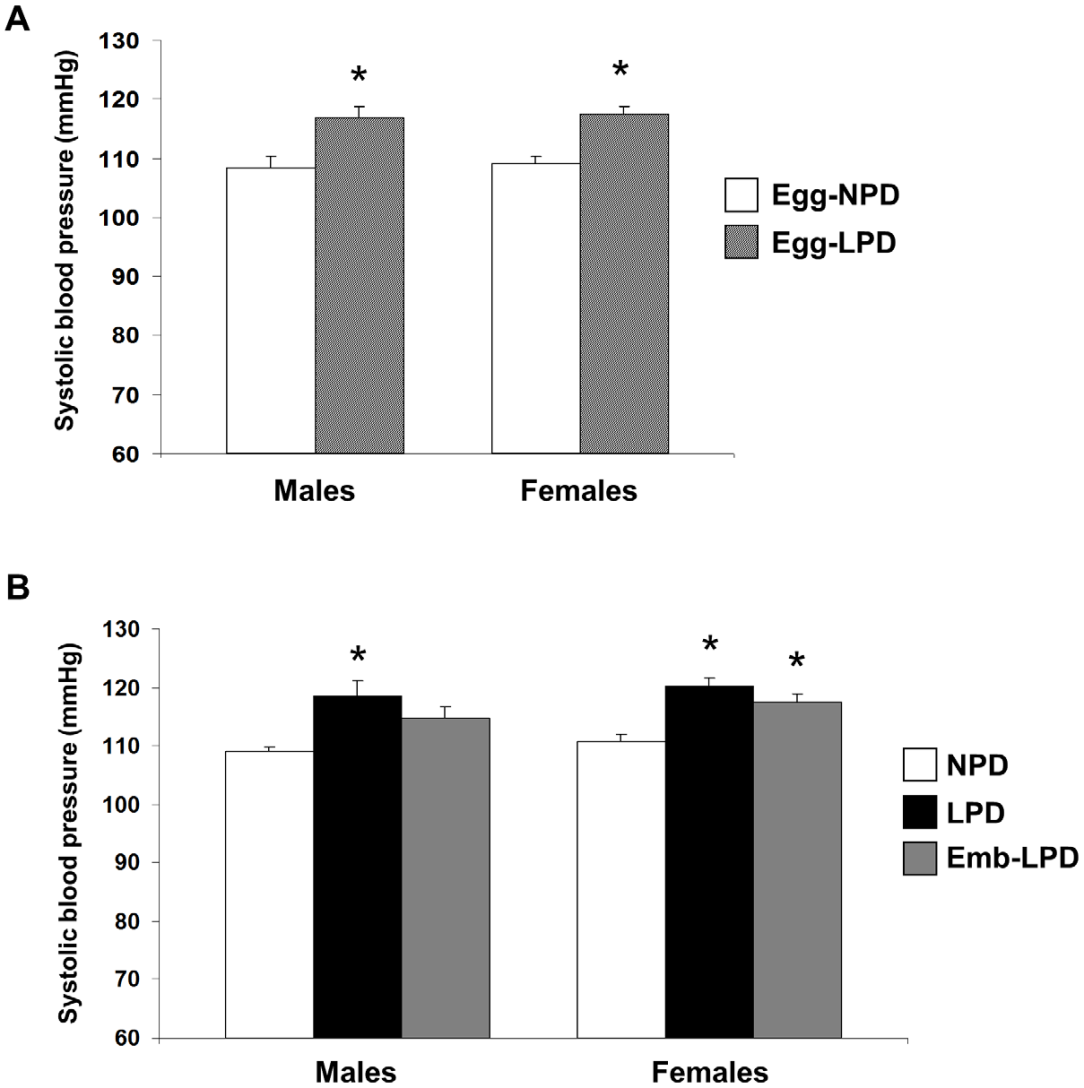
Offspring systolic blood pressure in response to maternal diet. Mean systolic blood pressure at 52 weeks of age from (A) offspring fed NPD or LPD exclusively during oocyte maturation (Egg-NPD and Egg-LPD respectively), or (B) fed NPD or LPD throughout gestation, or LPD exclusively during preimplantation development (Emb-LPD). Values are means for 9–13, 11–12, 9–12, 8–10 and 9–11 Egg-NPD, Egg-LPD, NPD, LPD and Emb-LPD males and females respectively ± SEM; *P<0.01.

Egg-LPD males and females displayed elevated SBP at 52 weeks of age when compared to Egg-NPD offspring (Fig. 3A; P = 0.002 and <0.001 respectively). LPD males and females and Emb-LPD females had elevated SBP compared to NPD males and females (Fig. 3B; P = 0.013, 0.002 and 0.011 respectively). All outcomes were independent of body weight and litter size effects.

### Serum glucose and insulin levels

No differences were observed between Egg-NPD and Egg-LPD offspring, or between NPD, LPD and Emb-LPD offspring in mean serum glucose or insulin levels (Table 6).

**Table 6:** Fasted offspring serum insulin and glucose concentrations.

		NPD		LPD		Emb-LPD		Egg-NPD		Egg-LPD	
		*Mean*	*SEM*	*Mean*	*SEM*	*Mean*	*SEM*	*Mean*	*SEM*	*Mean*	*SEM*
**Insulin (ug/L)**	Males	2.400	0.190	2.160	0.161	2.546	0.153	1.990	0.088	2.202	0.115
	Females	2.004	0.155	1.842	0.056	1.972	0.135	1.991	0.122	1.882	0.051
**Glucose (mg/dL)**	Males	174.767	21.51	214.501	19.031	169.887	18.523	166.634	12.341	156.187	12.626
	Females	153.665	8.989	149.36	7.317	162.561	17.852	150.881	9.386	151.341	8.937

Values are means ± SEM for 9–13, 11–12, 9–12, 8–10 and 9–11 Egg-NPD, Egg-LPD, NPD, LPD and Emb-LPD males and females respectively.

### Organ allometry

Egg-LPD males had a reduced lung weight ratio (when expressed as percentage of body weight) compared to Egg-NPD males (P<0.001) and an increased carcass weight (body weight minus combined weight of organs and fat pads; P = 0.04) (Table S1a–d). No other differences in mean organ weight, organ: body weight percentages or fat pad weights were observed between Egg-NPD and Egg-LPD offspring.

**Figure 4:**
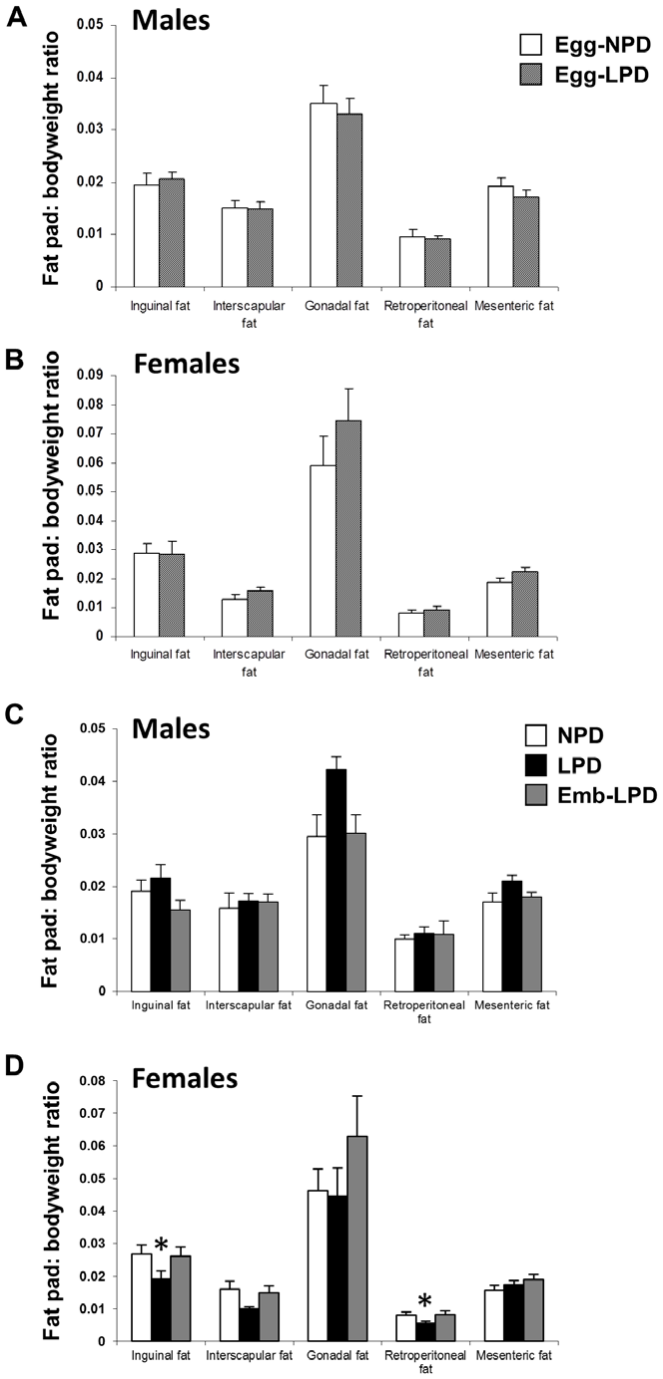
Mean offspring fat pad: body weight ratios. Mean fat pad: body weight ratio from (A) male or (B) female offspring fed NPD or LPD exclusively during oocyte maturation (Egg-NPD and Egg-LPD respectively), or (C) male or (D) female offspring fed NPD or LPD throughout gestation, or LPD exclusively during preimplantation development (Emb-LPD). Values are means for 9–13, 11–12, 9–12, 8–10 and 9–11 Egg-NPD, Egg-LPD, NPD, LPD and Emb-LPD males and females respectively ± SEM; *P<0.02.

No differences in individual organ weights were observed between NPD, LPD and Emb-LPD offspring (Table S1a–d). However, LPD females had reduced inguinal and retroperitoneal fat pad weights compared to NPD females (P = 0.024 and 0.029 respectively). These differences remained when the data were expressed as fat pad: body weight ratios (Fig. 4D; inguinal pad ratio P = 0.011, retroperitoneal pad ratio P = 0.016). Analysis of Emb-LPD female combined fat pad weight revealed a non-significant increase of 22% over NPD females (data not shown), whilst mean combined organ and carcass weights were only altered by <2%. Similarly, LPD females displayed <1% change in total organ weight and only a 5.5% decrease in carcass weight, but a non-significant decrease of 20% in total adiposity, when compared to the NPD females.

In our previous analysis of offspring development at 6 months, we identified positive associations between perinatal weight (at 3 weeks of age) and adult health markers, specific to the timing and duration of maternal diet [26]. To determine whether these, and additional associations, were still evident at 1 year of age, we correlated perinatal weight with weight, SBP, metabolic and adiposity parameters at 1 year. Interestingly, weight at 3 weeks of age was still a positive determinant of weight at 1 year in Emb-LPD offspring (r = 0.614, P = 0.004; Table 7), but not for any other treatment group.

**Table 7:** Correlations of adult body weight (at 52 weeks of age) with perinatal weight (at 3 weeks of age), adult mean total fat pad weight and serum insulin and glucose levels.

Correlations with Weight at 52 weeks; r values	Weight at 3 Weeks	Total Fat Pad Weight	Serum Insulin	Serum Glucose
NPD	0.366	0.613*	0.578*	0.085
LPD	0.162	0.774*	0.631*	0.589*
Emb-LPD	0.614*	0.632*	0.691*	−0.513*
Egg-NPD	−0.085	0.657*	0.551*	0.215
Egg-LPD	−0.088	0.468*	0.456*	0.180

Values are means for 9–13, 11–12, 9–12, 8–10 and 9–11 Egg-NPD, Egg-LPD, NPD, LPD and Emb-LPD males and females respectively;

*p<0.03.

Weight at 1 year was significantly positively correlated with total fat pad mass and with serum insulin levels in all treatment groups (Table 7; P<0.05). However, glucose levels only correlated with body weight in the LPD and Emb-LPD groups, and in opposite directions (r = 0.589, −0.513 and P = 0.039 and 0.021 respectively). Total fat pad weight was a poor indicator of serum insulin and glucose levels, only being significant for insulin in Egg-NPD and for glucose in Emb-LPD groups (r = 0.456 and −0.493, P = 0.05 and 0.045 respectively). Similarly, serum insulin levels only correlated with glucose levels in LPD offspring (r = 0.588, P = 0.016). Interestingly, SBP did not correlate with any of the parameters measured within the study for any of the treatment groups (data not shown).

### Fat pad gene expression

**Figure 5:**
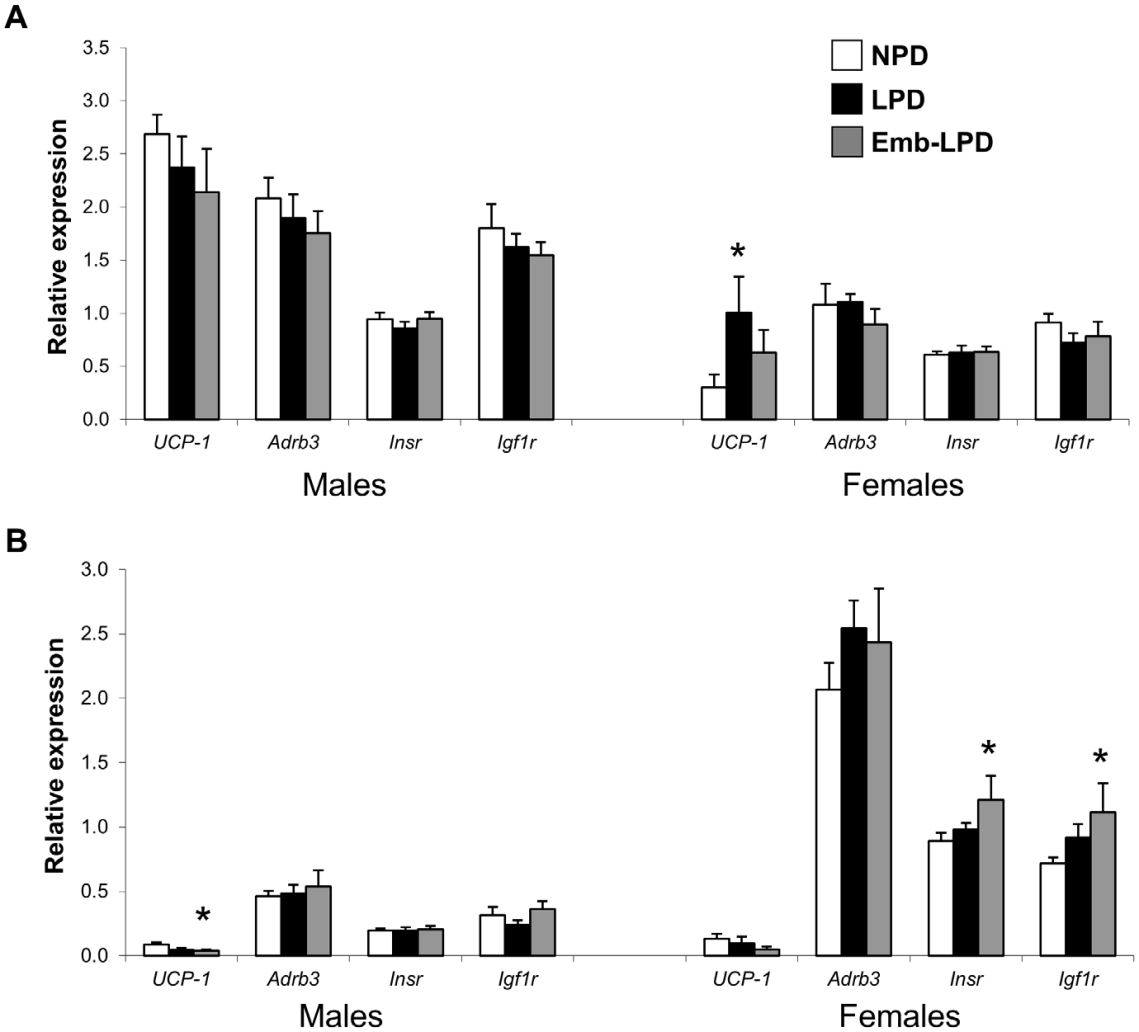
Fat pad regulatory gene expression profiles. Relative real-time RT-PCR expression values from male and female (A) interscapular and (B) retroperitoneal fat tissues. Values are means for 9–12, 8–10 and 9–11 NPD, LPD and Emb-LPD males and females respectively ± SEM; *P≤0.05.

As significant differences in body and fat pad weights were observed between NPD, LPD and Emb-LPD offspring, the expression profiles of several key adipose regulating genes from IBAT and white adipose tissue (WAT; from the retroperitoneum) were determined by RTqPCR. Emb-LPD females had higher expression of *Insr* and *Igf1r* in retroperitoneal fat compared to NPD females (P = 0.006 and 0.005 respectively; Fig. 5). LPD females had elevated *Ucp1* in IBAT compared to NPD females (P = 0.05), whilst Emb-LPD males displayed a lower expression of *Ucp1* in retroperitoneal fat (P = 0.029).

Positive correlations of adult (52 weeks of age) weight and IBAT *Ucp1* and *Adrb3* expression were observed in NPD and LPD offspring (P≤0.01), however, the strength of this association was reduced within Emb-LPD offspring (Table 8). Similarly, negative correlations of retroperitoneal fat *Insr* and *Igf1r* were observed in NPD and LPD offspring (P≤0.01), however, only *Igf1r* expression correlated negatively in Emb-LPD offspring (Table 8). Combined fat pad weight and serum insulin and glucose levels were poorer correlates of adipose gene expression patterns except in LPD offspring where significant positive and negative correlations of both insulin and glucose were observed with IBAT and WAT respectively (P<0.05; Table 9, 10 and 11).

**Table 8:** Correlations (r values) of body weight at 52 weeks of age with relative real-time RT-PCR expression values from interscapular brown adipose tissue (IBAT) and retroperitoneal white adipose tissue (WAT).

	IBAT		WAT	
	*Ucp1*	*Adrb3*	*Insr*	*Igf1r*
**NPD**	0.774*	0.623*	−0.657*	−0.739*
**LPD**	0.614*	0.689*	−0.652*	−0.792*
**Emb-LPD**	0.396	0.622*	−0.44	−0.608*

Values are means for 9–12, 8–10 and 9–11 NPD, LPD and Emb-LPD males and females respectively;

*p<0.05.

**Table 9:** Correlations (r values) of total fat pad weigh with relative real-time RT-PCR expression values from interscapular brown adipose tissue (IBAT) and retroperitoneal white adipose tissue (WAT).

	IBAT		WAT	
	*Ucp1*	*Adrb3*	*Insr*	*Igf1r*
**NPD**	0.092	0.255	−0.062	−0.204
**LPD**	0.338	0.516*	−0.099	−0.324
**Emb-LPD**	−0.066	0.051	0.109	−0.029

Values are means for 9–12, 8–10 and 9–11 NPD, LPD and Emb-LPD males and females respectively;

*p<0.05.

**Table 10:** Correlations (r values) of serum insulin levels with relative real-time RT-PCR expression values from interscapular brown adipose tissue (IBAT) and retroperitoneal white adipose tissue (WAT).

	IBAT		WAT	
	*Ucp1*	*Adrb3*	*Insr*	*Igf1r*
**NPD**	0.414	0.244	−0.43	−0.455
**LPD**	0.549*	0.691*	−0.528*	−0.634*
**Emb-LPD**	0.388	0.595*	−0.442	−0.583*

Values are means for 9–12, 8–10 and 9–11 NPD, LPD and Emb-LPD males and females respectively;

*p<0.05.

**Table 11:** Correlations (r values) of serum glucose levels with relative real-time RT-PCR expression values from interscapular brown adipose tissue (IBAT) and retroperitoneal white adipose tissue (WAT).

	IBAT		WAT	
	*Ucp1*	*Adrb3*	*Insr*	*Igf1r*
**NPD**	0.013	−0.159	−0.129	0.128
**LPD**	0.510*	0.418	−0.597*	−0.598*
**Emb-LPD**	0.060	−0.095	−0.105	0.089

Values are means for 9–12, 8–10 and 9–11 NPD, LPD and Emb-LPD males and females respectively;

*p<0.05.

## Discussion

Development of the metabolic syndrome has been identified as a significant risk factor for future adult health. Metabolic syndrome components (obesity, insulin and glucose insensitivity and hypertension) have been identified in offspring derived from a range of different maternal gestational nutritional challenges [32]. The period in development during which offspring growth, metabolic and cardiovascular physiology displays maximal susceptibility to programming is widely debated. Gestational LPD in the rodent programs changes in offspring growth which perpetuate into adult life [33], [34]. Interestingly, feeding these offspring hypercaloric or high fat diets postnatally [35], or the suckling of rat pups from lean mothers by obese dams [36], subsequently programs offspring obesity, hypertension, insulin resistance and serum leptin and glucose levels, highlighting the sensitivity of the early perinatal period. However, our current study is, to our knowledge, the first to demonstrate that maternal periconceptional protein undernutrition in the mouse programmes growth, SBP and adiposity homeostasis in aging offspring.

This study aimed to determine whether altered adult phenotypes evident at 6 months, induced through maternal periconceptional diet [25], [26], persisted into aging mice. Our first major observation was that the enhanced growth phenotype in Emb-LPD females at 6 months was maintained until 1 year of age despite a smaller sample size. Interestingly, by 1 year, LPD females had become lighter than NPD females, a phenotype not observed at 6 months. Analysis of individual correlates revealed that weight at 3 weeks of age was still a significant predictor of adult weight at 1 year for Emb-LPD offspring. We also observed significant positive interactions of adiposity (combined fat pad weight) with weight at 1 year in all treatment groups. These findings demonstrate how early life factors (such as size and weight) can be more predictive of, and associate strongly with, adult disease susceptibility, a central concept of the Developmental Origins of Health and Disease (DOHaD) hypothesis [37].

To gain further mechanistic insights into the changes in body weight observed, we assessed whole body adiposity and gene expression profiles in white (retroperitoneal; WAT) and brown adipose tissue (interscapular; IBAT). Whilst LPD females had significantly reduced inguinal and retroperitoneal fat pad weights, no significant changes were observed in Emb-LPD female adiposity when compared to NPD females. Analysis of gene expression patterns revealed Emb-LPD females had elevated *Insr* and *Igf1r* expression in WAT, but reduced IBAT *Ucp1* expression. In contrast, LPD females displayed increased IBAT *Ucp1* expression. LPD females also displayed strong positive associations of insulin and glucose levels with IBAT *Ucp1* and *Adrb3* expression, whilst negative associations with WAT *Insr* and *Igf1r* expression. WAT has a predominant role in energy storage in the form of triacylglycerol. Stimulation of the insulin (*Insr*) and IGF-1 (*Igf1r*) receptors induces glucose transporter translocation, increasing intracellular glucose levels. In contrast, adrenergic signalling, through the β3-adrenoceptor (*Adrb3*) in IBAT upregulates expression of inner mitochondrial membrane UCP-1 [38], uncoupling the proton electrochemical gradient and liberating heat [39]. Elevated IBAT *Ucp1* in LPD females, in addition with serum metabolite (glucose and insulin) correlations (positive IBAT *Ucp1* and *Adrb3*, negative WAT *Insr* and *Igf1r*) are suggestive of an energy liberating phenotype. In contrast, elevated WAT *Insr* and *Igf1r* expression coupled with reduced IBAT *Ucp1* expression in Emb-LPD females is suggestive of an energy storage phenotype. Nutritional programming of offspring adipose gene expression has been reported. In the rat, manipulation of maternal diet during gestation has been shown to alter adipose carbohydrate, lipid, and protein metabolism gene expression patterns [8], alter WAT expression profiles to resemble that of IBAT [39] and upregulate mRNA expression of angiotensinogen and adiponectin [40].

The DOHaD hypothesis proposes that offspring metabolic homeostatic levels are set during fetal development in direct response to maternal nutritional cues. However, subsequent mismatch between predicted and actual nutritional levels increases adult disease risk [37]. Previously [26], we speculated that in response to the significant changes in maternal nutritional environment following Emb-LPD [30], the preimplantation embryo initiates mechanisms to enhance nutrient retrieval and maintain growth. However, the subsequent mismatch between pre- and postimplantation nutrition results in these adaptations becoming maladaptive, driving enhanced fetal and postnatal growth and adult hypertension. Our current findings, that Emb-LPD females appear to up-regulate energy storing and down-regulate energy-utilising pathways in adipose tissue, suggest that an enhanced nutrient retrieval/storage phenotype is still evident at 1 year, and still associates with hypertension. Interestingly, LPD females displayed an opposite adipose phenotype, suggestive of increased energy utilisation and reduced adiposity. It therefore seems that embryonic adaptations made in response to preimplantation LPD were appropriate for the remainder of gestation, preventing excessive fetal and perinatal growth, but become maladaptive during later adult life, programming enhanced energy utilisation, significant weight and adiposity loss as well as hypertension.

Adipose tissue and the adipokines it secrets also has a role in the development of cardiovascular disease. Factors such as angiotensinogen, adiponectin, leptin, angiotensin-converting enzyme and plasminogen activator inhibitor-1 (PAI-1) are all secreted from adipose tissue and have been implicated in the development of cardiovascular disease in the offspring [41]. However, whilst no direct correlation between fat pad weight and adult SBP was observed for any group, potential influences of such factors on offspring cardiovascular regulation cannot be ruled out. Alternatively, additional factors may also have influenced SBP regulation. Previously, we demonstrated that resistance arteries from male Egg-LDP, LPD and Emb-LPD groups displayed impaired endothelial-dependent and independent vasodilatation and elevated angiotensin-converting enzyme activity [25], [26]. Similar impairments in vascular dynamics and cardiac function have been observed in offspring from rat dams fed LPD throughout gestation [14], [42] and sheep exposed to maternal undernutrition during pre- and periconceptional periods [43]. Studies have also revealed impairments in nephrogenesis in rat offspring from LPD dams programming hypertension [44], whilst aortic stiffness, reduced vascular smooth muscle cell number, endothelial dysfunction and decreased kidney Na^+^-K^+^-ATPase activity have been reported following maternal over nutrition [45]. Additional investigations would be necessary for identifying the mechanisms involved in the elevated SBP observed within the present study.

Offspring glucose and insulin homeostasis appear equally sensitive to maternal gestational nutrition. Both over- and under-nutrition result in offspring hyperphagia, adiposity and insulin resistance [46], [47], and appear age and sex dependent. As offspring age, initial insulin sensitivity is replaced with insulin resistance in later adulthood [47], [48]. In rats, 20 week old male offspring from LPD dams were found to be insulin resistant and hyperinsulinemic whilst female offspring of the same age were not [49]. Similarly, Ozanne et al., showed that fifteen month old males from LPD rat dams displayed impaired glucose tolerance whilst females displayed similar insulin level results to controls in glucose tolerance tests [50]. However, no significant differences in offspring serum glucose or insulin levels were observed between any treatment groups. As with our SBP data, additional studies into glucose/insulin tolerance as well as pancreatic structure and function would be necessary to fully determine offspring homeostatic capacity.

As these and our own data highlight, sex specific phenotypic responses are often observed following maternal dietary manipulation. Female offspring from mice fed high-fat diets prior to gestation become hypertensive and hypercholesterolemic and have reduced locomotor activity [51] but not males. In the rat, the use of maternal LPD exclusively during the preimplantation period elevated systolic blood pressure only in male offspring [30], whilst a maternal low-sodium diet reduced fetal growth and increased blood pressure only in females [52]. One explanation for the sex specific effects could be the differences in sex hormones which may influence fetal/postnatal development affecting adult disease risk. The progression of renal injury has been linked to androgen levels in males [53] whilst a protective role has been shown for estrogen [54]. Recently, studies have also identified sex specific patterns of gene expression in human placentas [55] as well as those collected from mouse dams fed diets of differing fat content [56]. In addition, differences between the stages exposed within the current study (post fertilisation development versus gamete maturation), and as such potential influence of the presence (post-fertilisation) or absence (pre-fertilisation) of the paternal genome during the dietary manipulation period could also differentially affect fetal and postnatal development.

In conclusion, our results are the first to demonstrate the effects of maternal periconceptional nutrition on adult body weight, cardiovascular physiology and adiposity regulation in aging animals. Our findings reveal subtle but significant differences in central regulatory processes influencing adult body weight and adipose tissue development and function dependent upon the duration and stage in development during which maternal nutrition is manipulated. Whilst the evidence from human and other animal model studies support the premise that early sub-optimal environmental conditions can alter adult physiology and disease risk, the mechanisms underlying these long-term alterations are not yet fully understood. Further research is therefore essential for the identification of fundamental factors important for prevention of metabolic diseases in adulthood.

## Supporting Information

Table S1 **Mean organ weight and organ: body weight ratios for male and female offspring.** Values are means ± SEM for 9–13, 11–12, 9–12, 8–10 and 9–11 Egg-NPD, Egg-LPD, NPD, LPD and Emb-LPD males and females respectively; * P≤0.04.
(DOCX)
