# Supplementary material for: Maternal Periconceptional and Gestational Low Protein Diet Affects Mouse Offspring Growth, Cardiovascular and Adipose Phenotype at 1 Year of Age
Source: PLoS One. 2011 Dec 15;6(12):e28745. doi: 10.1371/journal.pone.0028745 (PMC3240629; doi:10.1371/journal.pone.0028745)
Supplement: Table S1 — Mean organ weight and organ: body weight ratios for male and female offspring. Values are means ± SEM for 9–13, 11–12, 9–12, 8–10 and 9–11 Egg-NPD, Egg-LPD, NPD, LPD and Emb-LPD males and females respectively; * P≤0.04. (DOCX) [file pone.0028745.s001.docx]

Table S1

1. Male organ weights (g)

|  | **Body weight** | | **Carcass weight** | | **Liver** | | **Left kidney** | | **Right kidney** | | **Heart** | | **Lung** | | **Brain** | | **Cerebellum** | |
| --- | --- | --- | --- | --- | --- | --- | --- | --- | --- | --- | --- | --- | --- | --- | --- | --- | --- | --- |
|  | **Mean** | **SEM** | **Mean** | **SEM** | **Mean** | **SEM** | **Mean** | **SEM** | **Mean** | **SEM** | **Mean** | **SEM** | **Mean** | **SEM** | **Mean** | **SEM** | **Mean** | **SEM** |
| **Egg-NPD males** | **49.23** | **2.41** | **39.88** | **1.76** | **2.209** | **0.135** | **0.407** | **0.013** | **0.404** | **0.012** | **0.243** | **0.009** | **0.262** | **0.011** | **0.478** | **0.019** | **0.056** | **0.003** |
| **Egg-LPD males** | **51.07** | **1.64** | **43.57*** | **0.96** | **2.397** | **0.099** | **0.389** | **0.017** | **0.406** | **0.017** | **0.257** | **0.011** | **0.253** | **0.012** | **0.462** | **0.008** | **0.055** | **0.002** |
| **NPD males** | **50.95** | **2.36** | **43.69** | **1.78** | **2.368** | **0.121** | **0.380** | **0.019** | **0.398** | **0.017** | **0.236** | **0.008** | **0.248** | **0.006** | **0.452** | **0.009** | **0.053** | **0.001** |
| **LPD males** | **51.48** | **1.42** | **41.07** | **0.96** | **2.462** | **0.212** | **0.384** | **0.014** | **0.400** | **0.016** | **0.255** | **0.012** | **0.270** | **0.015** | **0.463** | **0.009** | **0.060** | **0.002** |
| **Emb-LPD males** | **49.41** | **1.27** | **41.36** | **1.54** | **2.287** | **0.113** | **0.389** | **0.017** | **0.398** | **0.019** | **0.226** | **0.006** | **0.259** | **0.011** | **0.461** | **0.008** | **0.055** | **0.002** |

1. Male organ weight as a percentage of body weight

|  | **Carcass weight** | | **Liver** | | **Left kidney** | | **Right kidney** | | **Heart** | | **Lung** | | **Brain** | | **Cerebellum** | |
| --- | --- | --- | --- | --- | --- | --- | --- | --- | --- | --- | --- | --- | --- | --- | --- | --- |
|  | **Mean** | **SEM** | **Mean** | **SEM** | **Mean** | **SEM** | **Mean** | **SEM** | **Mean** | **SEM** | **Mean** | **SEM** | **Mean** | **SEM** | **Mean** | **SEM** |
| **Egg-NPD males** | **82.00** | **0.600** | **4.540** | **0.182** | **0.849** | **0.042** | **0.845** | **0.049** | **0.508** | **0.032** | **0.543** | **0.025** | **1.030** | **0.040** | **0.120** | **0.005** |
| **Egg-LPD males** | **82.70** | **0.600** | **4.560** | **0.191** | **0.738** | **0.028** | **0.765** | **0.029** | **0.487** | **0.013** | **0.478*** | **0.015** | **0.877** | **0.027** | **0.104** | **0.005** |
| **NPD males** | **82.30** | **0.600** | **4.600** | **0.221** | **0.731** | **0.032** | **0.767** | **0.026** | **0.470** | **0.020** | **0.496** | **0.022** | **0.916** | **0.055** | **0.107** | **0.006** |
| **LPD males** | **80.30** | **0.600** | **4.770** | **0.269** | **0.750** | **0.019** | **0.780** | **0.020** | **0.499** | **0.020** | **0.525** | **0.018** | **0.910** | **0.033** | **0.117** | **0.005** |
| **Emb-LPD males** | **82.60** | **0.700** | **4.600** | **0.175** | **0.789** | **0.048** | **0.805** | **0.043** | **0.457** | **0.018** | **0.521** | **0.016** | **0.932** | **0.028** | **0.111** | **0.006** |

1. Female organ weights (g)

|  | **Body weight** | | **Carcass weight** | | **Liver** | | **Left kidney** | | **Right kidney** | | **Heart** | | **Lung** | | **Brain** | | **Cerebellum** | |
| --- | --- | --- | --- | --- | --- | --- | --- | --- | --- | --- | --- | --- | --- | --- | --- | --- | --- | --- |
|  | **Mean** | **SEM** | **Mean** | **SEM** | **Mean** | **SEM** | **Mean** | **SEM** | **Mean** | **SEM** | **Mean** | **SEM** | **Mean** | **SEM** | **Mean** | **SEM** | **Mean** | **SEM** |
| **Egg-NPD females** | **43.09** | **1.72** | **34.47** | **1.08** | **1.944** | **0.100** | **0.236** | **0.012** | **0.240** | **0.012** | **0.182** | **0.006** | **0.250** | **0.013** | **0.490** | **0.011** | **0.063** | **0.002** |
| **Egg-LPD females** | **45.79** | **2.00** | **33.51** | **0.85** | **2.145** | **0.136** | **0.223** | **0.008** | **0.231** | **0.010** | **0.196** | **0.008** | **0.250** | **0.008** | **0.481** | **0.005** | **0.061** | **0.002** |
| **NPD females** | **40.79** | **1.18** | **33.58** | **0.48** | **1.846** | **0.063** | **0.197** | **0.008** | **0.214** | **0.007** | **0.182** | **0.007** | **0.238** | **0.004** | **0.467** | **0.006** | **0.059** | **0.001** |
| **LPD females** | **39.04** | **1.72** | **31.71** | **1.32** | **1.773** | **0.112** | **0.193** | **0.006** | **0.205** | **0.006** | **0.188** | **0.006** | **0.238** | **0.006** | **0.480** | **0.011** | **0.058** | **0.002** |
| **Emb-LPD females** | **42.91** | **2.12** | **32.98** | **1.34** | **1.825** | **0.072** | **0.209** | **0.009** | **0.216** | **0.009** | **0.180** | **0.006** | **0.235** | **0.009** | **0.471** | **0.006** | **0.058** | **0.002** |

1. Female organ weight as a percentage of body weight

|  | **Carcass weight** | | **Liver** | | **Left kidney** | | **Right kidney** | | **Heart** | | **Lung** | | **Brain** | | **Cerebellum** | |
| --- | --- | --- | --- | --- | --- | --- | --- | --- | --- | --- | --- | --- | --- | --- | --- | --- |
|  | **Mean** | **SEM** | **Mean** | **SEM** | **Mean** | **SEM** | **Mean** | **SEM** | **Mean** | **SEM** | **Mean** | **SEM** | **Mean** | **SEM** | **Mean** | **SEM** |
| **Egg-NPD females** | **80.80** | **1.800** | **4.540** | **0.185** | **0.558** | **0.036** | **0.568** | **0.040** | **0.430** | **0.022** | **0.588** | **0.028** | **1.200** | **0.079** | **0.154** | **0.012** |
| **Egg-LPD females** | **76.90** | **1.800** | **4.900** | **0.278** | **0.513** | **0.019** | **0.529** | **0.019** | **0.449** | **0.015** | **0.574** | **0.021** | **1.130** | **0.033** | **0.142** | **0.006** |
| **NPD females** | **81.00** | **10.000** | **4.530** | **0.132** | **0.487** | **0.026** | **0.532** | **0.028** | **0.448** | **0.018** | **0.587** | **0.019** | **1.040** | **0.120** | **0.131** | **0.015** |
| **LPD females** | **82.20** | **1.100** | **4.570** | **0.177** | **0.505** | **0.025** | **0.538** | **0.026** | **0.493** | **0.025** | **0.624** | **0.030** | **1.260** | **0.050** | **0.152** | **0.009** |
| **Emb-LPD females** | **79.40** | **1.000** | **4.310** | **0.138** | **0.496** | **0.022** | **0.514** | **0.024** | **0.428** | **0.020** | **0.560** | **0.029** | **1.120** | **0.055** | **0.137** | **0.007** |
